# Animal Models of Cerebral Palsy: Hypoxic Brain Injury in the Newborn

**Published:** 2015

**Authors:** Mark Daniel WILSON

**Affiliations:** Department of Medicine, Melbourne University, St Vincent’s Hospital Melbourne, Victoria, Australia

**Keywords:** Neurodevelopmental disorder, Prenatal hypoxia, Cerebral palsy, Murine model

## Abstract

**Objective:**

Hypoxic insults are implicated in the spectrum of fetal disorders, including cerebral palsy (CP). In view of the major contribution of intrapartum risk factors and prematurity to subsequent neurological morbidity and mortality in humans, this study aimed to clarify the pathophysiology of brain injury, especially

periventricular white matter damage (WMD), that occur in utero to the immature and near-term fetal CNS.

**Materials & Methods:**

An evaluation of the resulting neurological and behavioural phenotype in the newborn was performed by utilising a battery of neurobehavioural tests, including the Morris water-maze and the open-field test, followed by cerebral MRI and histopathology.

**Results:**

This study used a murine model to examine the deleterious effects of WMD brought about by cerebral hypoxia-ischemia (HI) and the characteristic features of CP in mice. Murine models have proven themselves valuable in the area of experimental neuroscience.

**Conclusion:**

Hypoxia-treated mice were observed to demonstrate a significant neurofunctional deficit compared with sham mice on two behavioral measures. Indeed, different brain regions, including the sensorimotor cortex, the striatum, and the hippocampus were noticeably damaged after HI insult, as determined by both

MRI and histopathology. These results, albeit qualitative in nature, appear to support the pre-existing finding that the long-term neurofunctional outcome in animal subjects with CP is strongly associated with the anatomical extent and pattern of cerebral damage as determined by both delayed neuroimaging and

histopathology.

## Introduction

The aim of this study was to produce irreversible fetal brain damage in an animal model that mimics the lesions associated with CP observed in the human fetus and neonate. Furthermore, an evaluation of the resulting neurological and behavioural phenotype in the newborn was made in relation to its usefulness as a model of CP. It was hypothesized that irreversible fetal brain damage brought about by cerebral hypoxia-ischemia produces neurological and behavioural deficits, including many of the symptoms of CP. These neurological and behavioural deficits were then assessed qualitatively with a battery of neurobehavioural tests. Cerebral HI is one of the main causes of brain injury in neonates, with a risk of neurological outcomes including CP, epilepsy or death (1). Given the great plasticity of the developing brain, functional measures of injury are likely to be more clinically relevant. The lack of a suitable fetal animal model that reproduces the motor deficits of CP has hampered definition of the mechanisms underlying the condition. Most previous studies have employed models of cerebral HI in postnatal animals (2). However, a significant mortality was typically observed or the animals recovered completely and neurobehavioral deficits were difficult to elicit experimentally. As mentioned previously, the brains of rats and mice are thought to be as mature at 7 to 10 days as third trimester human fetuses, making the delineation of neurodevelopmental outcomes a practical and worthwhile task. This study therefore utilized a murine model to examine the effects of WMD brought about by cerebral HI. Three-day-old (P3) mice of both genders were used in this experiment; 20 mice (10 neonatal mice and 10 adult mice) were subjected to HI. The right common carotid artery was ligated under isoflurane anesthesia. The mice were then allowed to recover for a period of 1-2 hours. The mice were then subjected to 8% O2 balanced with N2 for 20 minutes at 37°C to induce hypoxia. After hypoxic exposure, the pups were returned to their mothers. For the purposes of this experiment, control animals consisted of ten age-matched (adult mice) and ten sham (no HI) mice. The use of these controls took into account the mechanisms underlying post-ischaemic damage and how it differs between young and older mice (3). For example, the immaturity of the blood-brain barrier may affect the nature of the sustained damage, as well as changes in cerebral blood flow during the first few weeks of life (4). These factors are important for the cerebral response to injury. The use of sham mice (no HI) removed the source of cerebral injury, thereby allowing direct examination of cerebral HI in the treated neonatal mice. At eight weeks after HI, neurofunctional outcome was assessed with a battery of neurobehavioural tests, including the Morris water-maze and the open-field test, followed by cerebral MRI and histopathology. The Morris water-maze was performed to assess spatial learning and memory for function of the hippocampus. The open-field test was undertaken to assess gross cerebral structural integrity. These two tests were performed in mice subjected to HI in the neonatal period, as well as in the two control groups. All mice were given a chance to practice the Morris water-maze before definitive results were obtained. After a twoday rest period, mouse behaviour was monitored in the open-field apparatus. This device consists of a plastic chamber of around 40x40x30 cm, which is criss-crossed with infrared beams spaced 1.00-1.25 cm apart to record the location of the mice and the traveled path length. All mice were tested for 60 minutes, in a standardised fashion, during which various behavioural criteria (gait and basic decision making, among other variables) was digitally recorded. It is postulated that the long-term neurofunctional outcome in mice after HI is strongly associated with an anatomical pattern of cerebral damage (5,6). At 10 weeks after HI, all mice were subjected to a T2-weighed magnetic resonance imaging (MRI) study. Imaging was performed on a Bruker AVANCE 400WB spectrometer. During the imaging experiment, the mice were anesthetized with an isoflurane/air gas mixture via a nose pump. Images were then obtained using a 2-dimensional multislice spin echo (SE) sequence. Twelve coronal slices were acquired, covering the entire brain. After neurobehavioral testing and MRI study, all mice were euthanized in the standard fashion with a barbiturate. The brains were taken and fixed in 10% formaldehyde at approximately 5°C for 24-36 hours, and then mounted in paraffin wax. Multiple 10-µm coronal sections were obtained and the degree of cerebral atrophy or scarring was qualitatively assessed in relation to the proportion of normal brain tissue. In each cerebral section, histological examination of the hippocampus, the striatum, and associated thalamic areas, including the hypothalamus, was made.

## Results

 Ten weeks after HI, the MRI studies revealed a combination of ipsilateral brain atrophy alone or with porencephalic cyst formation and ipsilateral ventriculomegaly. The damaged cerebral regions on the MRI images appeared brighter than the darker normal brain tissue, given that a T2-weighed study was utilized. Moreover, the sensorimotor cortex, the striatum, and the hippocampus were noticeably damaged after HI insult, as determined by both MRI and histopathology. Hypoxia-treated adult mice were observed to demonstrate a significant neurofunctional deficit compared with sham mice on the two behavioral measures. However, HI-treated neonatal mice demonstrated an even more pronounced neurofunctional deficit. Therefore, one would expect neurobehavioral assessment of mice subjected to HI insult to reveal a strong correlation between degree of brain injury and neurofunctional deficit. In relation to the Morris watermaze, the HI-treated mice, especially the neonates, displayed a significant delay in locating the platform. The anatomical extent of brain damage, assessed by both MRI and histopathology, was associated with the degree of navigational memory deficit and impaired spatial reasoning. The more severely affected HI-treated mice, especially the neonates, had significant difficulty with the Morris water-maze. In fact, these mice were generally unable to traverse this environment, and demonstrated impairment of movement and coordination. Interestingly, the open-field test demonstrated that exploratory behaviour of HI-treated mice was not significantly different compared with sham mice. However, it was determined that the HI-treated neonatal mice exhibited significantly reduced ambulatory velocity, reduced number of ambulatory episodes, and increased ambulatory distance (increased superfluous movements whilst moving from one point to the next) compared with either HI-treated adult mice or sham mice. These results, albeit qualitative in nature, appear to support the pre-existing finding that the long-term neurofunctional outcome in animal subjects with CP is strongly associated with the anatomical extent and pattern of cerebral damage as determined by both delayed neuroimaging and histopathology.

## Discussion

**Defining Cerebral Palsy**

 Cerebral palsy is the most common physical disability in childhood, occurring in 2-2.5 per 1000 live births in developed countries (7). The prevalence of CP has not changed over the last 40 years, despite a fourfold drop in both perinatal and maternal mortality. Cerebral palsy is not a single disease, but rather a group of neurological impairments characterized by abnormal control of movement or posture. Abnormalities in brain development and acquired non-progressive cerebral white matter lesions are implicated as causes of CP. The motor disorders characteristic of CP are often accompanied by disorders of sensation, perception, cognition and verbal communication, as well as epilepsy and myriad other musculoskeletal abnormalities (7,8). The association of CP with white matter damage in very premature infants led to investigations of the significance of intrauterine infection to adverse neurological outcomes. The central paradigm is that maternal infection, occurring during specific gestational periods, leads to both a maternal and fetal inflammatory response, thus contributing to preterm delivery, WMD, and other neurological sequelae (9). In order to determine the broad effects of maternal immunity and adverse effects on the offspring, additional studies are required that focus on these interrelationships. For the purposes of this study, WMD secondary to hypoxia was the focus of investigation. White matter damage is the most frequently observed brain lesion in preterm infants. The exact aetiology is yet to be fully elucidated, however cerebral hypoperfusion and subsequent ischemia has been implicated as a main risk factor (4). One group compared the neuropathological outcome, including the effect on oligodendrocytes, astrocytes, and microglia following either systemic asphyxia or endotoxemia in fetal sheep at midgestation (10). This experiment resulted in microglia activation and amplification in the white matter, damaged astrocytes, and loss of oligodendrocytes. These results show that the white matter at midgestation is particularly sensitive to injury following both systemic asphyxia and endotoxemia. Asphyxia induced lesions in both white and subcortical grey matter is associated with microglia activation; endotoxemia resulted in selective WMD and inflammation (9,10).

**Fig 1:**
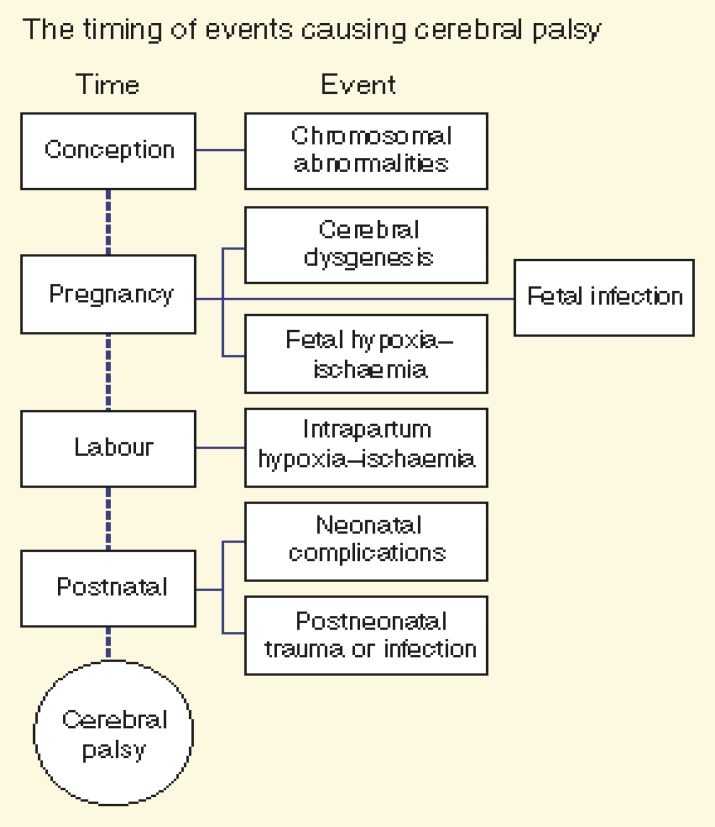
The timing of events causing CP in the newborn; it can be seen that multiple events occurring during the course of normal development can lead to CP (4).

**Animal Models and the Aetiology of Cerebral Palsy**

Animal models have greatly assisted in understanding the mechanisms of brain injury underlying CP. Still, no animal model replicates every aspect of the condition in humans. Despite this inherent limitation, animal models provide a practical means of obtaining fundamental information. As mentioned above, the neuropathology underlying CP includes WMD and HI. The type of damage of particular importance is periventricular leukomalacia; white matter damage is also associated with varying degrees of germinal matrix hemorrhage with intraventricular extension, and injury to the cortex, basal ganglia and thalamus (11). Periventricular leukomalacia consists of diffuse injury of deep cerebral white matter, with or without focal necrosis and a local inflammatory response. Recent pathological work focusing on human postmortem tissue has examined the role of free radical injury, cytokine toxicity, especially in regard to the epidemiologic association of periventricular leukomalacia with fetomaternal infection, and excitotoxicity in the development of periventricular leukomalacia (12). This work has focused on premyelinating oligodendrocytes, which predominate in periventricular regions during the period of vulnerability to periventricular leukomalacia (24-34 postconceptional weeks). Premyelinating oligodendrocytes are the targets of free radical injury, as determined by immunocytochemical markers of lipid peroxidation and protein nitration. This developmental susceptibility can be partly attributed to a relative deficiency of superoxide dismutase, the enzyme that neutralizes superoxide in developing white matter (13). Microglia, which respond to cytokines and to bacterial products such as lipopolysaccharide via Toll-like receptors, are augmented in periventricular leukomalacia white matter and also contribute to cellular damage (14,15). Several cytokines, including tumor necrosis factor- and interleukins 2 and 6, as well as interferon- , have been demonstrated in periventricular leukomalacia. One pivotal study suggests a role for glutamate receptors and glutamate transporters in periventricular leukomalacia based on expression in human developing oligodendrocytes (15). Germinal matrix hemorrhage, with or without intraventricular hemorrhage, occurs in premature infants and can coexist with periventricular leukomalacia. Studies in germinal matrix tissue have focused on maturation-based vascular factors, such as morphometry and expression of molecules related to the intricate structure of the blood-brain barrier (4). Graymatter injury, seen more commonly in term infants, includes cortical infarcts and “status marmoratus” (12). The cortical injury overlying periventricular leukomalacia is of interest because it serves as a possible substrate for the cognitive difficulties seen in children with CP (16). 

**Fig 2:**
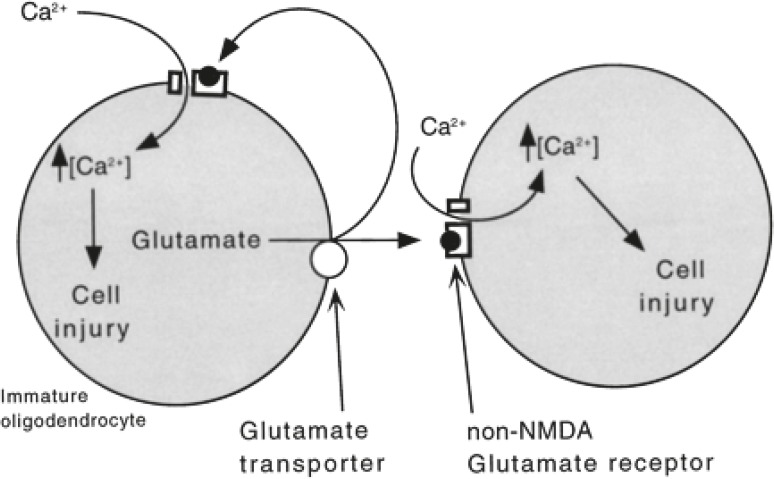
A model of ischemic cell death in immature oligodendrocytes; ischemia leads to the release of glutamate from cells via reverse transport. The buildup of xtracellular glutamate resulted in the gating of Ca2+-permeable non-NMDA glutamate receptors resulting in the influx of Ca2+ and cell death, with some glutamate feeding back on the cell that released it (13).

Progress in understanding the pathogenesis of periventricular WMD requires the development of animal models that are relevant to the unique physiology of the preterm fetal human brain, and that replicate the major neurological and behavioral features of human CP (17). In this domain of experimental physiology, the sheep is the most extensively studied animal model. The neurodevelopment of the preterm sheep fetus (0.65-0.70 gestation) is comparable to that of the preterm human fetus between 24 and 28 weeks. The size of the fetal sheep allows long-term instrumentation so that well-defined insults can be accurately studied with reliable measurements of blood flow and metabolism in cerebral white matter (18). Recent developments in this area have focused on the role of cerebral HI and vulnerable oligodendrocyte progenitors in the pathogenesis of periventricular WMD in the immature sheep fetus. Doppler ultrasonography and other effective imaging modalities have further defined cerebral blood flow measurements in utero (19,27). It has been determined that ovine white matter maturation between 90 and 120 days’ gestation, as defined by immunohistochemical localization of oligodendrocyte lineage-specific antibodies (18). Indeed, it is now established that there is considerable spatial and temporal heterogeneity in oligodendrocyte maturation in the immature periventricular white matter. Furthermore, oligodendrocyte maturation in the 90 to 105-day fetal sheep closely coincides with that of the preterm infant during the high-risk period for WMD. Additionally, the immature state of the 90 to 105-day fetal periventricular white matter is an optimal and dynamic developmental window to study the role of cellular-maturational factors in the pathomechanics of WMD (16-18). On the basis of these findings, there are significant, demonstrable advantages of the instrumented fetal sheep as a model of CP in humans, especially in relation to WMD. Others, however, have proposed that a better understanding of the spatiotemporal events in prenatal brain development for different animal models is indispensible for the proper delineation of the precise neurodevelopmental impact of in utero infections and the associated WMD in humans (19-21). This will also help to better characterize the critical neuroimmunological mechanisms implicated in aberrant brain development following prenatal exposure to infection (20-22). Indeed, more attention needs to be given to the central role cerebral HI plays in human CP, principally because CP is often caused by injuries sustained during labour and birth (21-23). Prenatal HI of the developing brain has been strongly associated in the subsequent development of the hypertonic motor deficits of CP in premature and full-term infants who present with neonatal encephalopathy (19,20). Despite the impact of CP, there is no entirely suitable animal model that reproduces the hypertonia and motor disturbances of this disorder. To remedy this situation, a rabbit model of in utero placental insufficiency was established, in which hypertonia is accompanied by marked abnormalities in motor control (17). Preterm fetuses (67-70% gestation) were subjected to sustained global hypoxia. The dams survived and gave spontaneous birth. At postnatal day 1 (P1), the pups that survived were subjected to a sequence of neurobehavioral tests. Newborn pups of hypoxic groups displayed significant impairment in several tests of spontaneous locomotion, reflex motor activity, and the coordination of suck and swallow. Increased tone of the limbs at rest and with active flexion and extension were observed in the survivors of the preterm insult. Furthermore, histopathological studies identified a distinct pattern of acute injury to subcortical motor pathways that involved the basal ganglia and thalamus. Injury to the caudate putamen and thalamus at P1 was significantly correlated with hypertonic motor deficits in the hypoxic group. Antenatal HI at preterm gestation thus results in hypertonia and abnormalities in motor control. These findings provide a unique behavioral framework to define mechanisms and sequelae of perinatal brain injury from antenatal HI and provide the first animal model which displays hypertonic motor deficits at term birth that are consistent with those observed in CP (24-27). This study utilized a murine model to examine the pernicious effects of WMD brought about by cerebral HI and the characteristic features of CP in mammals. Indeed, rodents have proven to be extremely practical animal models in the realm of experimental neuroscience for myriad reasons. The brains of rats and mice are as mature at 7 to 10 days as third trimester human fetuses in relation to parameters such as number and volume of synaptic connections, neurochemical development, and cortical organization, so comparative studies yield considerable knowledge. The experiment detailed in this study elucidated long-term neuroanatomical and pathological changes as they correlate with neurofunctional deficits, including many of the features of CP in adult mice subjected to HI at a very early developmental stage. In rodents, the sensorimotor cortex, the striatum, and the hippocampus are predominantly damaged after HI insult, as revealed by both imaging and gross histopathological analysis. In relation to the study of cerebral MRI assessment of neonatal mice subjected to HI, there is an early evolution of neuroanatomical changes. According to these findings, the damaged area of the brain reaches a maximal extent at three to six hours after HI. Furthermore, all MRI-assessed cerebral changes were found within the affected hemisphere at all-time points. As it is now recognized, cerebral palsy is not a single disease entity, but a constellation of neurological impairments. Abnormalities in brain development and acquired non-progressive cerebral white matter lesions are implicated as causes of CP. Fundamental insults, including hypoxic-ischemic events, occurring during specific gestational periods, leads to both a potentiated maternal and fetal inflammatory response, which precipitates preterm labour, white matter damage and the resulting neurological deficits. In order to determine the broad effects of HI and subsequent WMD on the offspring, interdisciplinary and translational studies of a more quantitative nature are required to clarify causation of this important problem in clinical neurology.

## References

[1] Ikeda T, Mishima K, Yoshikawa T, Iwasaki K, Fujiwara M, Xia YX, Ikenoue T (2001). Selective and longterm learning impairment following neonatal
hypoxicischemic brain insult in rats. Behav Brain Res.

[2] Keogh JM, Badawi N (2006). The origins of cerebral palsy. Curr Opin Neurol.

[3] Sheldon RA, Sedik C, Ferriero DM (1998). Strain-related brain injury in neonatal mice subjected to
hypoxiaischemia. Brain Res.

[4] Muramatsu K, Fukuda A, Togari H, Wada Y, Nishino H (1997). Vulnerability to cerebral hypoxic-ischemic insult in neonatal but not in
adult rats is in parallel with disruption of the blood-brain barrier. Stroke.

[5] Northington FJ (2006). Brief update on animal models of hypoxic-ischemic encephalopathy and
neonatal stroke. Inst Lab Anim Res J.

[6] Xue R, Sawada M, Goto S, Hurn PD, Traystman RJ, van Zijl PCM, Mori S (2001). Rapid three-dimensional diffusion MRI facilitates the study of acute stroke
in mice. Magn Reson Med.

[7] MacLennan AH (1995). The origins of cerebral palsy – a consensus statement. MJA.

[8] Gomez R, Romero R, Ghezzi F, Yoon BH, Maxor M, Berry SM (1998). The fetal inflammatory response syndrome. Am J Obstet Gynecol.

[9] Hagberg H, Malard C (2005). Effect of inflammation on central nervous system development and
vulnerability. Curr Opin Neurol.

[10] Mallard C, Welin A, Peebles D, Hagberg H, Kjellmer (2003). White Matter Injury Following Systemic Animal Models of Cerebral Palsy:
Hypoxic Brain Injury in the Newborn Endotoxemia or Asphyxia in the Fetal
Sheep. Neurochemical Research.

[11] Rumpel H, Nedelcu J, Aguzzi A, Martin E (1997). Late glial swelling after acute cerebral hypoxia-ischemia in the neonatal
rat: a combined magnetic resonance and histochemical study. Pediatr Res.

[12] Folkerth RD (2005). Neuropathologic substrate of cerebral palsy. J Child Neurol.

[13] Fern R, Moller T (2000). Rapid ischemic cell death in immature oligodendrocytes: a fatal glutamate
release feedback loop. J Neurosci.

[14] Baumann N, Pham-Dinh D (2001). Biology of oligodendrocyte and myelin in the mammalian central nervous
system. Physiol Rev.

[15] Back SA, Luo NL, Borenstein NS, Levine JM, Volpe JJ, Kinney HC (2001). Late oligodendrocyte progenitors coincide with the developmental window of
vulnerability for human perinatal white matter injury. J Neurosci.

[16] Stigger F, Felizzola AL, Kronbauer GA (2011). Effects of fetal exposure to lipopolysaccharide, perinatal anoxia and
sensorimotor restriction on motor skills and musculoskeletal tissue: Implications for an
animal model of cerebral palsy. Exp Neurol.

[17] Derrick M, Luo NL, Bregman JC, Jilling T, Ji X, Fisher K, Gladson C, Beardsley DJ, Murdoch G, Back S, Tan S (2004). Preterm fetal hypoxia-ischemia causes hypertonia and motor deficits in the
neonatal rabbit: A model for human cerebral palsy?. J Neurosci.

[18] Back SA, Riddle A, Hohimer R (2006). Role of instrumented fetal sheep preparations in defining the pathogenesis
of human periventricular white-matter injury. J Child Neurol.

[19] Bell MJ, Hallenbeck JM, Gallo V (2004). Determining the fetal inflammatory response in an experimental model of
intrauterine inflammation in rats. Pediatr Res.

[20] Dammann O, Kuban KC, Leviton A (2002). Perinatal infection, fetal inflammatory response, white matter damage, and
cognitive limitations in children born preterm. Ment Retard Dev Disabil Res Rev.

[21] Derrick M, Drobyshevsky A, Ji X, Tan S (2007). A model of cerebral palsy from fetal hypoxia-ischemia. Stroke.

[22] Aden U, Dahlberg V, Fredholm BB, Lai LJ, Chen Z, Bjelke B (2002). MRI evaluation and functional assessment of brain injury after hypoxic
ischemia in neonatal mice. Stroke.

[23] Edgar JM, McLaughlin M, Yool D, Zhang SC, Fowler JH, Montague P (2004). Oligodendroglial modulation of fast axonal transport in a mouse model of
hereditary spastic paraplegia. J Cell Biol.

[24] Ten VS, Bradley-Moore M, Gingrich JA, Stark RI, Pinsky DJ (2003). Brain injury and neurofunctional deficit in neonatal mice with
hypoxic-ischemic encephalopathy. Behav Brain Res.

[25] Volpe JJ, Kinney HC, Jensen FE (2011). The developing oligodendrocyte: key cellular target in brain injury in the
premature infant. Int J Dev Neurosci.

[26] Zhu AH, Hu YR, Liu W, Gao F, Li J, X, Zhao LH, Chen G (2014). Systemic Evaluation of Hypoxic-Ischemic Brain Injury in Neonatal
Rats. Cell biochemistry and biophysics.

[27] van de Looij Y, Vasung L, Sizonenko SV, Hüppi PS (2014). MRI of animal models of developmental disorders and translation to human
imaging. Current opinion in neurology.

